# The Impact of Alcohol Consumption on Cardiovascular Health: Myths and Measures

**DOI:** 10.5334/gh.1132

**Published:** 2022-07-22

**Authors:** Monika Arora, Ahmed ElSayed, Birgit Beger, Pamela Naidoo, Trevor Shilton, Neha Jain, Kelcey Armstrong-Walenczak, Jeremiah Mwangi, Yunshu Wang, Jean-Luc Eiselé, Fausto J. Pinto, Beatriz M. Champagne

**Affiliations:** 1 Public Health Foundation of India, IN; 2 Alzaiem Alazhari University, SD; 3 European Heart Network, BE; 4 Heart and Stroke Foundation South Africa, ZA; 5 University of the Western Cape, ZA; 6 University of Western Australia, AU; 7 Curtin University, AU; 8 World Heart Federation, CH; 9 CCUL, University of Lisbon, PT; 10 CLAS Coalición América Saludable/Coalition for Americas’ Health, AR/US

**Keywords:** Alcohol, Alcohol Control, Cardiovascular Disease, CVD, Risk Factor, Public Health, Policy

## Abstract

Over the past several decades, the prevalence of cardiovascular disease (CVD) has nearly doubled, and alcohol has played a major role in the incidence of much of it. Alcohol has also been attributed in deaths due to infectious diseases, intentional and unintentional injuries, digestive diseases, and several other non-communicable diseases, including cancer.

The economic costs of alcohol-associated health outcomes are significant at the individual as well as the country level. Risks due to alcohol consumption increase for most cardiovascular diseases, including hypertensive heart disease, cardiomyopathy, atrial fibrillation and flutter, and stroke. The widespread message for over 30 years has been to promote the myth that alcohol prolongs life, chiefly by reducing the risk of coronary heart disease (CHD). Lack of universal advice and stringent policy measures have contributed towards increased uptake and easy availability of alcohol. The WHO has called for a 10% relative reduction in the harmful use of alcohol between 2013–2025. However, lack of investment in proven alcohol control strategies, as well as persistence of misinformation and industry interference, have hindered the efforts of public health professionals to make sufficient progress in reducing alcohol related harms and death.

**Figure:**
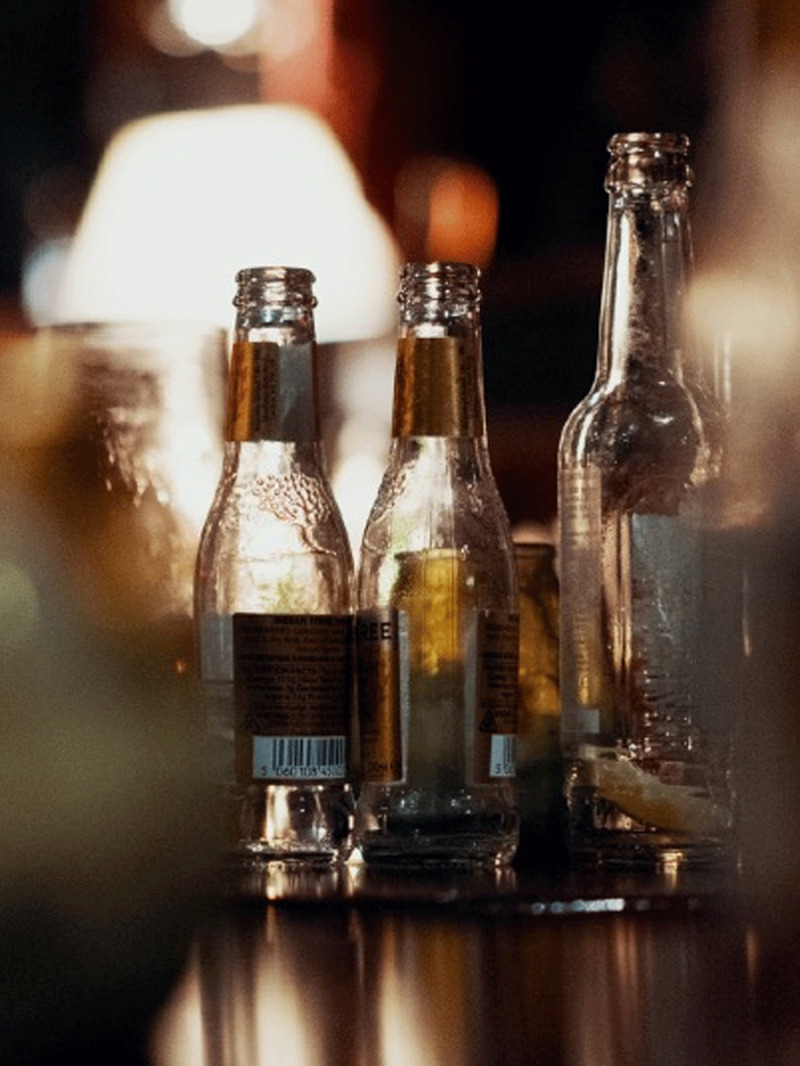


## Introduction

The prevalence of cardiovascular disease (CVD) has nearly doubled in the last two decades, with more than 500 million cases being reported in 2019 alone [1]. **More than 18.5 million individuals have died of CVD, making it the leading cause of global mortality and disability**. Preventable behavioural risk factors play a major role in incidence of CVD, including unhealthy diet, tobacco use, alcohol consumption, and low physical activity.

Alcohol is a psychoactive and harmful substance that has become a common accompaniment of social events in many parts of the world. In addition to being commonly interspersed with the social lifestyles of individuals, the use of alcohol exhibits a socio-economic inequity. **Individuals with low socio-economic status experience a disproportionately greater alcohol-attributable harm than individuals with high socio-economic status from similar or lower amounts of alcohol consumption** [2]. Furthermore, when compared by income groups, a higher overall burden of death was observed in lower and middle income countries compared to high-income countries [1].

Alcohol was targeted in the Sustainable Development Goals (SDGs) under SDG 3.5, which calls on countries to ‘strengthen prevention and treatment of substance abuse, including narcotic drug abuse and harmful use of alcohol’ [3]. This inclusion highlighted the role of alcohol as a development obstacle and its close association with many other SDGs and their targets. Alcohol adversely effects 14 out of 17 SDGs and 54 of the targets that make up the 2030 agenda [4].

With the unprecedented rise in the death and disability from alcohol-attributed CVD and other illnesses, it is imperative for countries and organizations to come together to impart a uniform, evidence-based message, and policy agenda for alcohol control. In a brief survey of Members of the World Heart Federation, 44.4% of respondents ‘strongly agreed’ and 51.9% of the respondents ‘agreed’ that national cardiology foundations/societies should publish guidelines and advocate for domestic policies to address the impact of alcohol on cardiovascular health. This policy brief:

- Summarises the epidemiology and burden of alcohol use.
- Explores the link between alcohol use and heart health.
- Abridges the alcohol ‘harm versus benefit’ debate.
- Presents recommendations for strengthening alcohol control globally.

**Figure:**
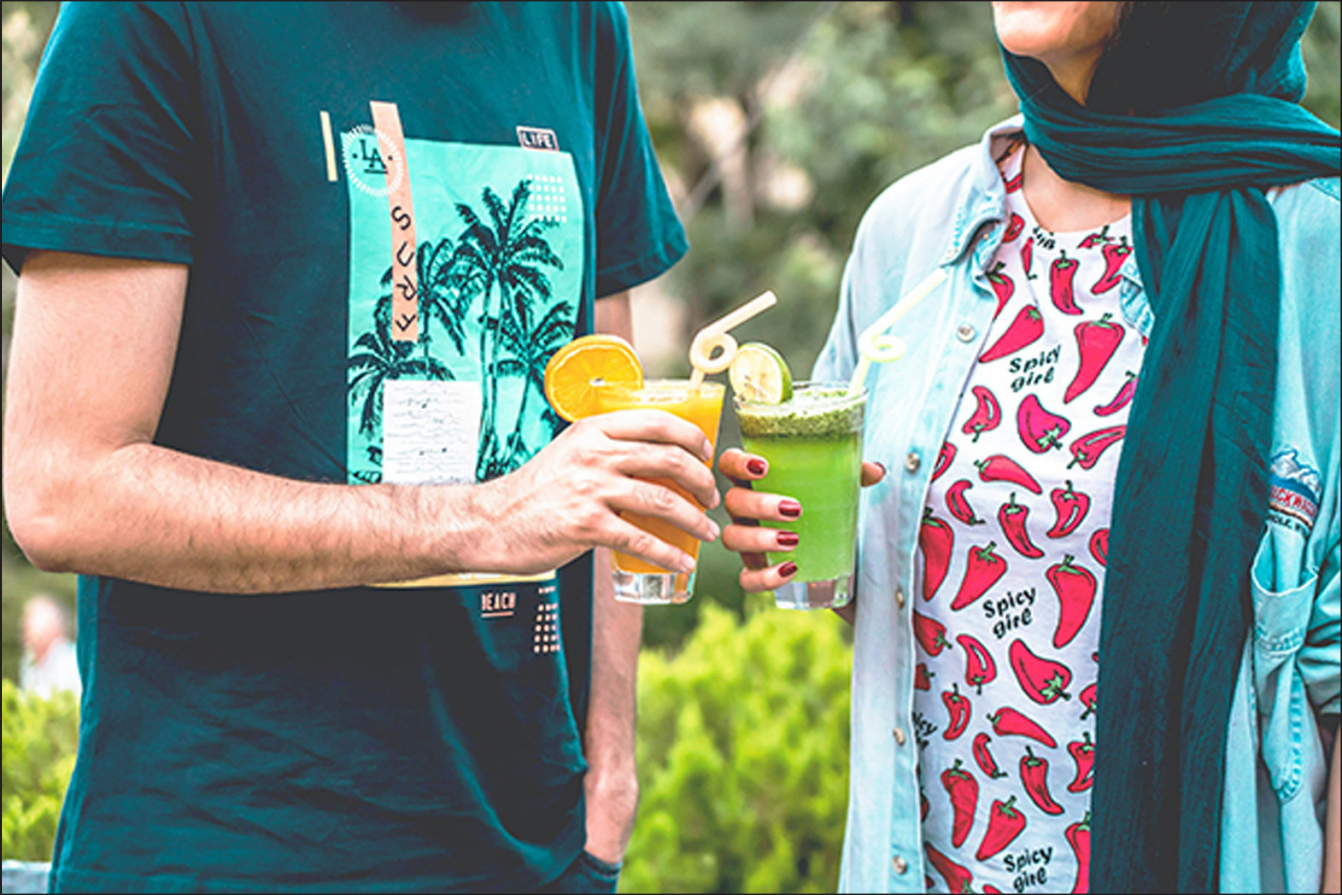

**Figure:**
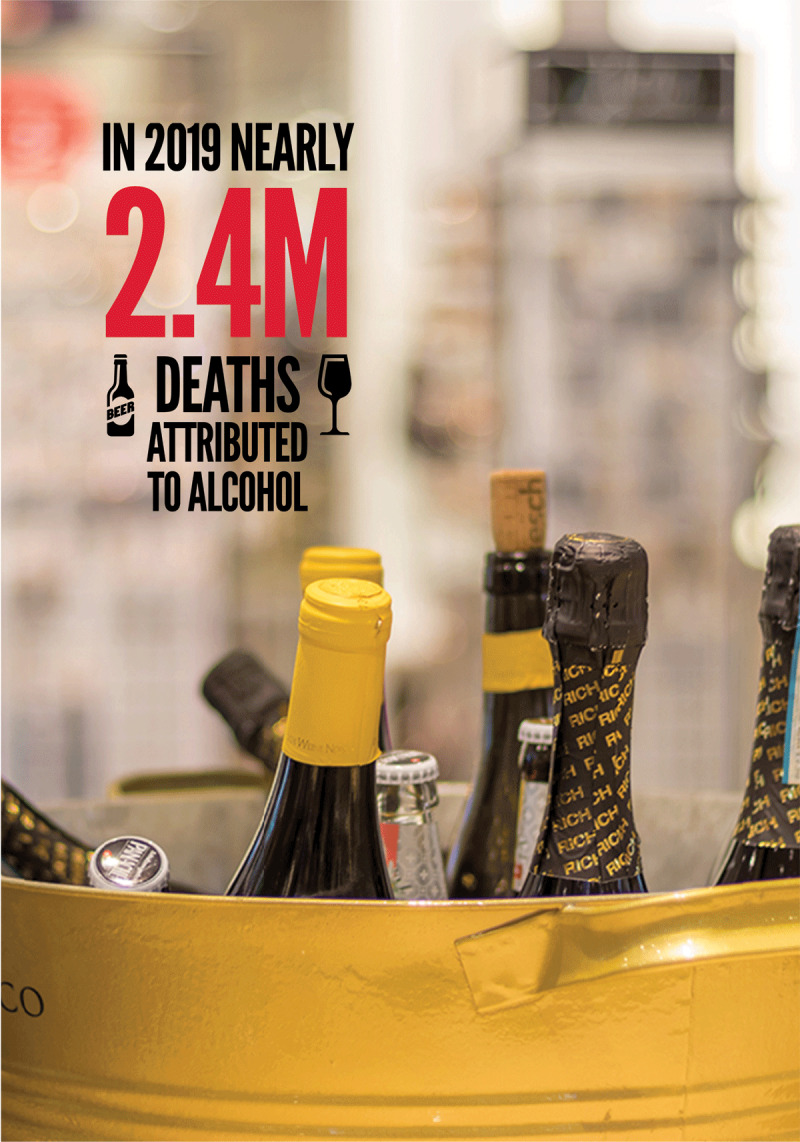

## Epidemiology and burden of alcohol use

Alcohol affects human physiology either through years of consumption, acute intoxication, or dependence [5]. It has been linked with approximately 230 ICD-10 (International Classification of Diseases, 10th edition) diseases, including 40 diseases that would not prevail without alcohol [6]. **Alcohol has been ascribed as a crucial factor in deaths due to infectious diseases, intentional and unintentional injuries, digestive diseases and several non-communicable diseases (NCD)** [7].

In 2016, high incidence of alcohol consumption was reported from high socio-demographic index (SDI) countries, where prevalence was 72% in females and 83% in males. In comparison, 8.9% of females and 20% of males were alcohol consumers in low and middle-income countries (LMICs) [5]. In 2019, nearly 2.4 million deaths were attributed to alcohol, accounting for 4.3% of all deaths globally.

Also, more than 92 million DALYs (Disability-adjusted life years) were lost due to alcohol in the same year [1]. Alcohol has been attributed in cancers of the oral cavity and pharynx, larynx, oesophagus, liver, stomach, breast, colon and rectum [8]. Even a small amount of alcohol has been linked with an increase in risk of breast cancer [9]. Women are less likely to consume alcohol than men; however, the use of alcohol may have more implications for women than men with respect to physical illnesses and more severe cognitive and motor impairment with a much lower alcohol exposure as compared to men [10].

Heavy drinkers have an increased risk of dying from liver cirrhosis [11] and there are a range of psychiatric disorders, particularly mood and anxiety disorders, associated with alcohol use [12]. Alcohol use has also been implicated in infectious diseases and poor health outcomes from such diseases. For example, heavy alcohol use (>40 g/day) causes a threefold increase in risk of active tuberculosis [13]. It is also known to exacerbate worse outcomes in HIV and tuberculosis patients due to decreased adherence to medicines, decreased health care utilisation, and increased HIV risk behaviours due to lack of sobriety [14]. Some countries have also found a four time increased risk of multimorbidity in individuals who drink alcohol [15].

Beyond the direct consequences on health of the drinker, the chronic use of alcohol is responsible for a significant societal impact and is linked with motor vehicle accidents, injuries, familial discord, and burden on a country’s criminal justice system, among other negative outcomes [16]. Children with parents who suffer from alcohol addiction have also been shown to exhibit higher rates of alcoholism in their life span [17].

Alcohol is also known to have a severe economic burden. Economic estimates from high income and middle income countries have shown that 1% of the gross domestic product (GDP) of such countries was spent on alcohol associated costs such as criminal justice costs and measures of lost productivity [18].

In a middle-income country such as India, it was estimated that direct and indirect costs from alcohol-related conditions would equate to USD 1.87 trillion between the years 2011 and 2050 [19], amounting to approximately 1.45% of the GDP per year of the Indian economy. This significant societal burden of alcohol includes the health system’s cost, out of pocket expenditure, and productivity losses.

**Figure:**
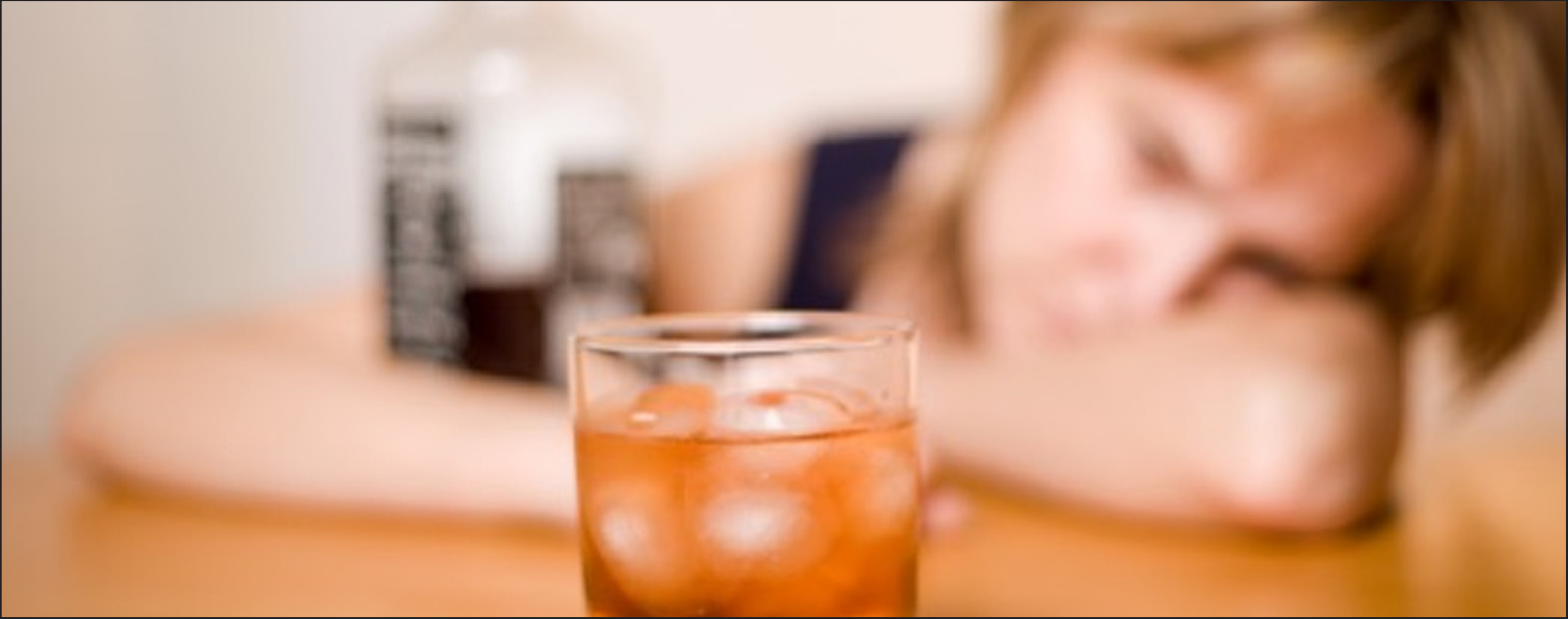


## Alcohol use and heart health

**Figure:**
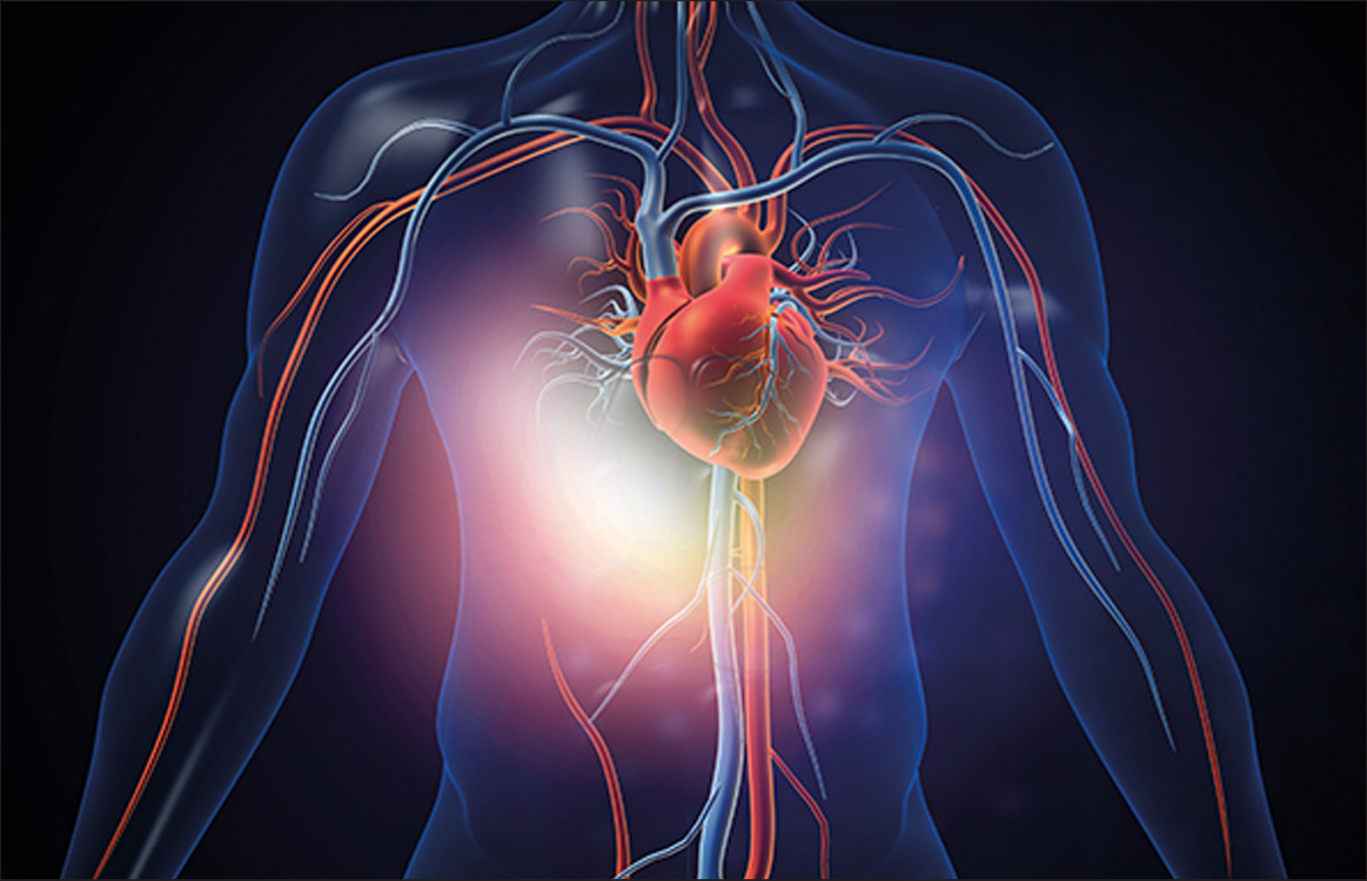


The Global Action Plan for Prevention and Control of NCDs, by the World Health Organization (WHO), calls for a 10% relative reduction in harmful use of alcohol between 2013–2025 [20].

There are multiple reasons that the belief that alcohol is good for cardiovascular health is no longer acceptable:

- Such evidence has been mostly based on observational studies.
- Comparisons to people who do not use alcohol are often confounded by social, cultural, religious, and medical reasons to not drink.
- Studies have been conducted in predominantly older (>55 years of age) and Caucasian populations.
- Some studies that show positive effects are funded by the alcohol industry [21].
- Alcohol use is often associated with other heart disease risk factors including tobacco use, access to health care, and other social determinants of health.
- No randomized controlled trials (RCTs) have confirmed health benefits of alcohol.

Alcohol increases the risk for hypertensive heart disease, cardiomyopathy, atrial fibrillation, flutter and strokes. Alcohol consumption (100 g/week) is linearly associated with a higher risk of stroke, heart failure, fatal hypertensive disease and fatal aortic aneurysm, and has a borderline elevation in the risk of coronary heart disease, as compared to those consuming between 0–25 g/week* [22]. It has been argued that people with moderate consumption and no binge episodes may appear to have a slightly lower risk of ischaemic heart disease (IHD), but the protective effect of moderate alcohol consumption for CVD has been challenged [23].

**Contrary to popular opinion, alcohol is not good for the heart.** This directly contradicts common and popular message that alcohol prolongs life, chiefly by reducing the risk of CVD.

The controversy over the role of low to moderate alcohol use and future heart attack relates to inconsistent results among the many studies on the topic. Historically, studies have shown a J-shaped distribution of outcomes. The lowest rates of heart attacks have been in those with low to moderate alcohol consumption and higher rates in those who did not drink or have high rates of alcohol consumption.

However, new research has challenged this interpretation by not confirming the J point relationship in Chinese [24] and Indian populations [25], where alcohol consumption is relatively lower, binge drinking is common and among people less than 55 years of age. Furthermore, there has been heterogeneity in the type and pattern of alcohol consumption in most parts of the world.

**Research in the latest decade has led to major reversals in the perception of alcohol in relation to health in general and CVD in particular.** These developments have prompted health authorities in a number of countries, e.g. the Netherlands [26], England [27] and Australia [28], to lower their recommended amount of alcohol for low-risk drinking.

The alcohol industry has also perpetuated misleading information about the benefits of drinking alcohol. This interference by the alcohol industry closely reflects the universally vilified activities of tobacco companies. Alcohol industries deceptively promote their products under the labels of ‘healthy’ and ‘safe’. Portrayal of alcohol in print and electronic media as necessary for a vibrant social life has diverted attention from the harms of alcohol use. Youth-targeted advertisement and encouraging alcohol as ‘heart-healthy’ have created a conducive environment for young adults to relate alcohol with ‘having a good time’. Contrary to this belief, evidence from all around the world exists to link alcohol with a range of non-communicable and infectious diseases.

* Alcohol consumption was roughly linearly associated with a higher risk of stroke (HR per 100 g per week higher consumption 1.14, 95% CI, 1.10–1.17), coronary disease excluding myocardial infarction (1.06, 1.00–1.11), heart failure (1.09, 1.03–1.15), fatal hypertensive disease (1.24, 1.15–1.33); and fatal aortic aneurysm (1.15, 1.03–1.28) as compared to those consuming between 0–25 g/week.

**Figure:**
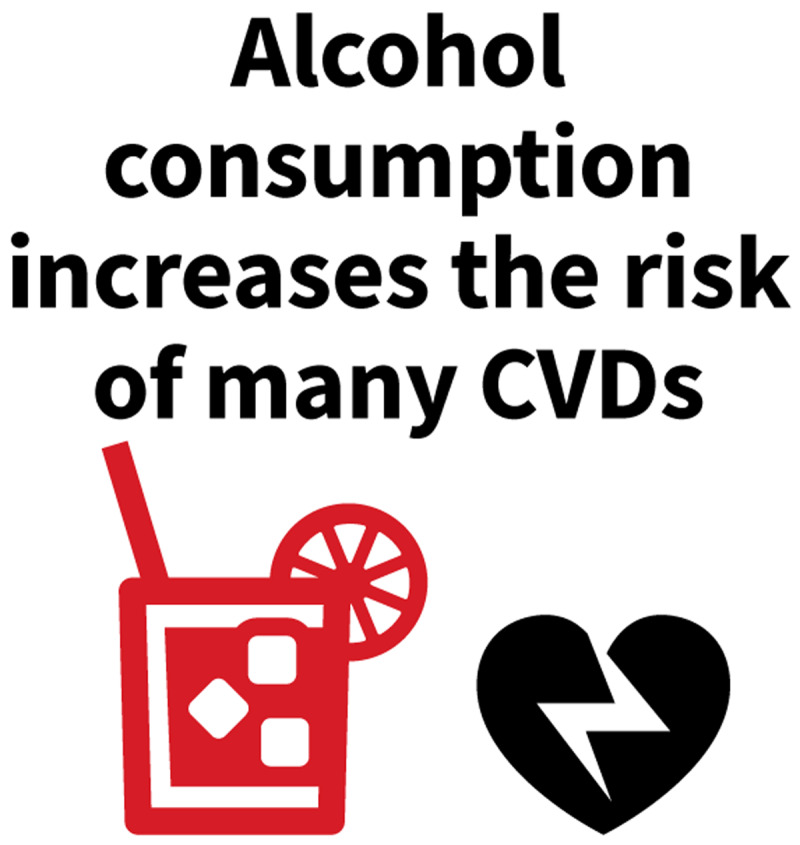


**Table 1:** Recommendations for alcohol use by target groups.


TARGET GROUP	WHAT IS THE RECOMMENDATION?	JUSTIFICATION FOR RECOMMENDATION

People living with cardiovascular diseases and other chronic illnesses	Abstinence	Alcohol increases the risk for hypertensive heart disease, cardiomyopathy, atrial fibrillation and flutter, and strokes. It is attributed in many other infectious and non-infectious diseases as well.

Pregnant women or those who are breastfeeding	Abstinence	Consumption of alcohol during pregnancy has been linked with Foetal Alcohol Syndrome [29], which is a combination of physical, behavioural and learning abnormalities in the new-born child. For mothers who are breastfeeding, no level of alcohol is safe for their babies.

Children and young people	Abstinence	Heavy drinking during adolescence and young adulthood is associated with lower neuro-cognitive functioning during the young adult years and particularly with impairment of attention and visio-spatial skills [30]. Early onset of alcohol use increases the risk of poor health outcomes, poor academic outcomes, and other problems during adolescence as well as a risk of developing an alcohol use disorder later on in life. The brain of an individual develops until the age of 25 years and alcohol use during this period negatively affects the brain [30].

Adults with no underlying health conditions	*For abstainers*: Not advised to start drinking *For drinkers*: There are no safe recommended levels of alcohol consumption – those who drink are advised to reduce their consumption for overall health.	Alcohol consumption negatively affects mental and physical health and is also linked with poorer quality of life and poverty. Even in smaller quantities, alcohol consumption can increase the risk of breast cancer. It can cause more severe motor and cognitive dysfunction in women at much lower levels of consumption than men. 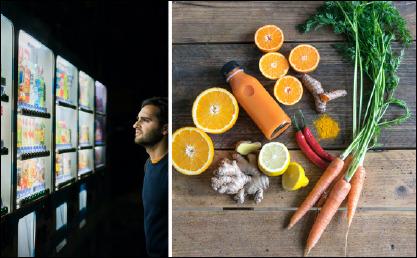


## Country case study [41]

After the dissolution of the Soviet Union and liberalisation in what is now the Russian Federation, alcohol consumption per capita increased to 20.4 litres by 2003, leading to significant alcohol-attributable mortality rates.

Per numerous studies, approximately 50% of all deaths among working-aged men were due to alcohol. In response, policy reforms were introduced to reduce alcohol consumption in Russia. These reforms included stricter penalties for drinking and driving, increases in excise taxes, setting minimum prices for some alcoholic products, restrictions on advertising, and restriction on alcohol availability. Strict alcohol control policies led to a significant reduction in alcohol-attributable morbidity and mortality. Between 2003 and 2016, alcohol consumption fell by 43%, alcohol dependence dropped, and a marked difference was observed in social impacts of alcohol (including suicide, homicide, and motor vehicle accidents) as well.

**Figure:**
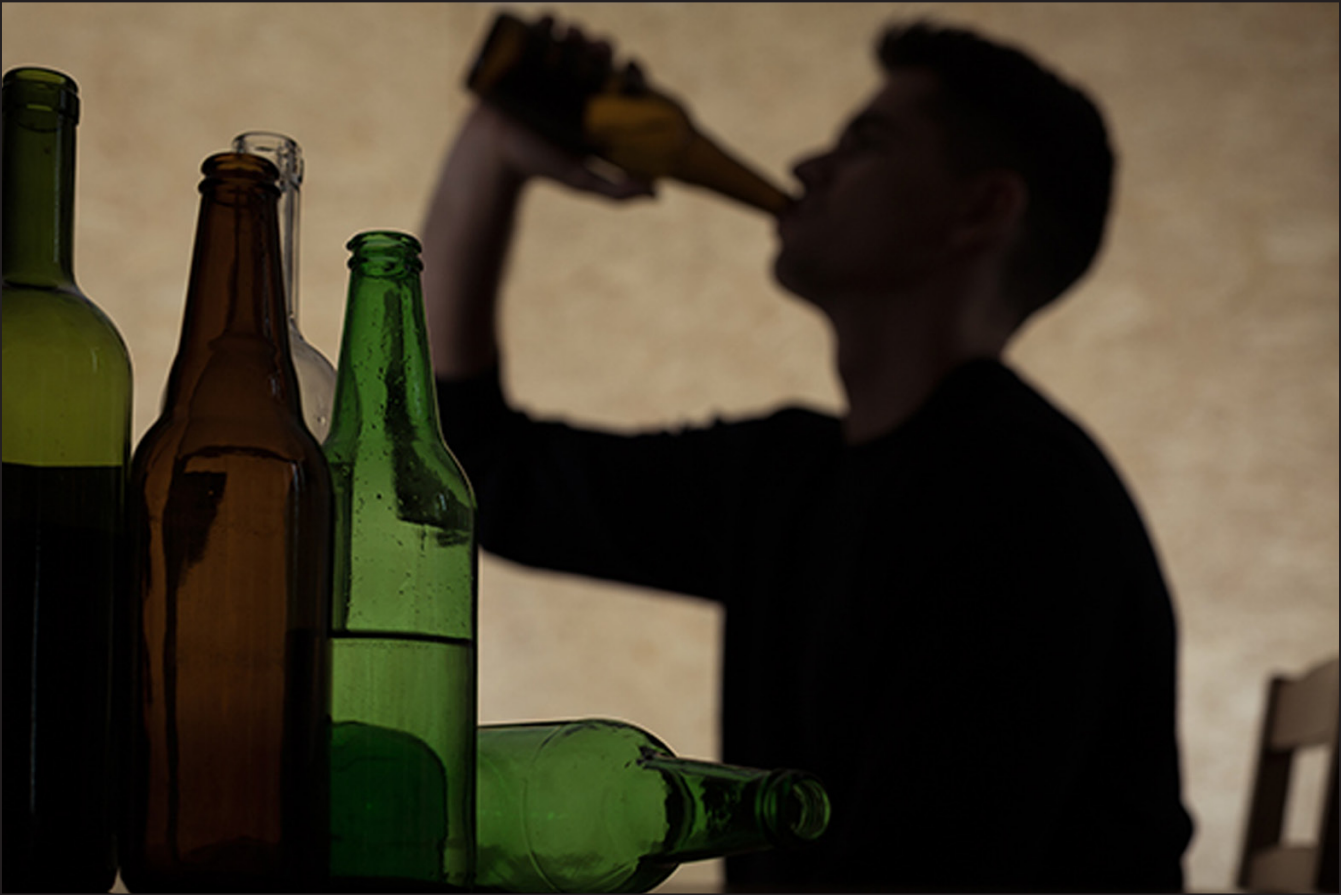


## Recommendations For Advocacy

**Table:** 


**National societies and organizations must play a central role in advocating for stricter alcohol control measures.** The voices of public evidence-based, public health-oriented agents are essential to ensuring the achievement of the Sustainable Development Goals and ultimately health equity. To begin with, all such actors should uniformly indicate the evidence on alcohol consumption and heart health. Ministries should implement strict regulatory measures to dissuade direct and indirect impacts of alcohol use (see Table 2). Finally, national cardiology societies and foundations can play the following roles for ensuring reduction in alcohol use and its related harms: Advocate for the adoption of WHO’s SAFER Guidelines in their local context [31] Call for strict regulation of alcohol products Advocate for minimum pricing of alcohol products Build capacity internally and among peers to promote cessation of alcohol use and reduce consumption among current users Promote community, national, and global best practices and materials, such as the PAHO “Live better, drink less” campaign, and advocate for their uptake [32] Communicate evidence on the harms of alcohol use, including that alcohol consumption increases the risk of many CVD Prioritise alcohol control in national agendas for health and support policy coherence between health and other sectors Facilitate screening for the use of alcohol and other substances as a part of risk mitigation during the health assessment of individuals visiting a health care centre Set the example of non-collaboration with the alcohol industry and/or its public relations groups.	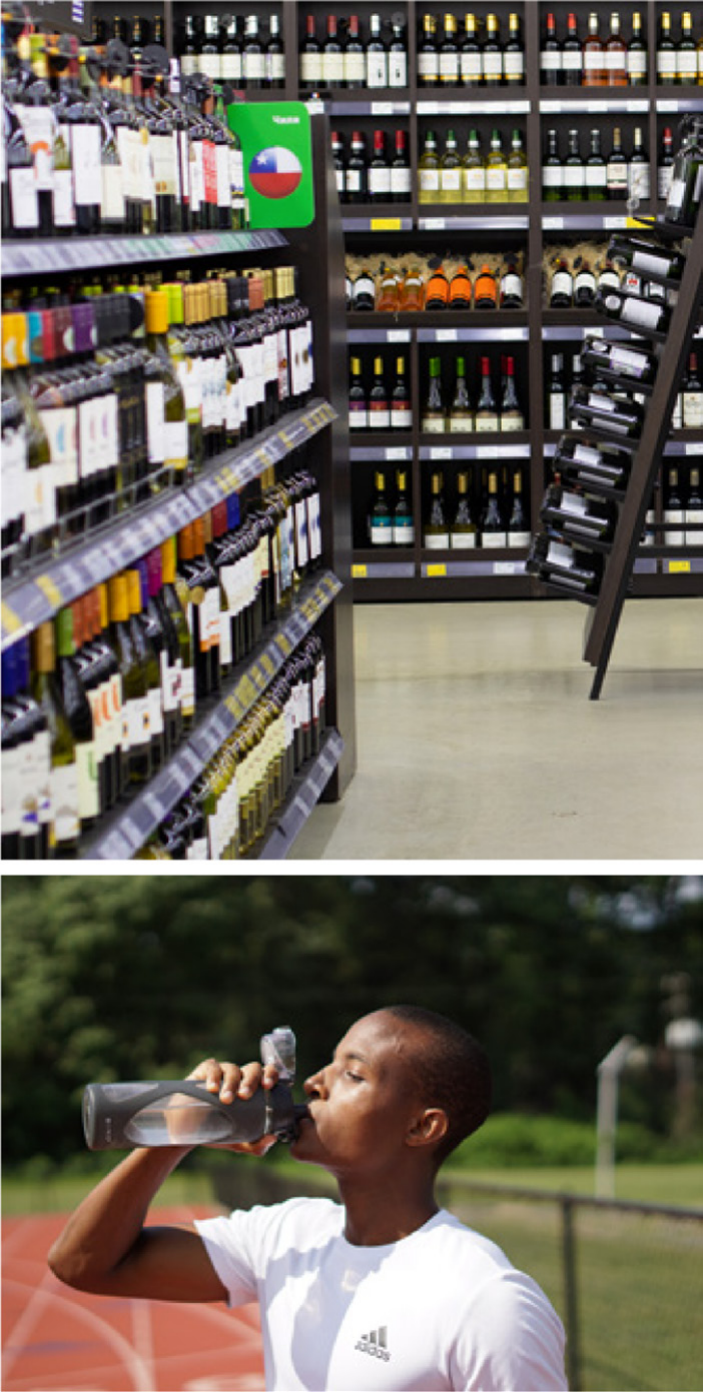


**Table 2:** Alcohol Policy – Best Practices.


	MEASURE	WHY IMPLEMENT IT?	COUNTRY BEST PRACTICE

**WHO SAFER Best Buys** [31]	Strengthen restrictions on alcohol availability	Restrictions on availability of unhealthy substances have proven to be cost-effective best-buy interventions for non-communicable disease prevention.	South Africa’s Liquor Amendment Bill prohibits licensed distributors from selling alcohol to unlicensed establishments [33].

	Advance and enforce drink driving countermeasures	Road traffic injuries are a rising and major cause of death and disability, especially among young adults. In high income countries, 20% of fatally injured drivers were found with excess blood alcohol content, and 33%-69% of road traffic fatalities in low-income countries were attributed to alcohol use.	A cluster of best practices were identified in Lithuania, where policies such as increase in excise taxes on alcoholic products, increase in legal minimum purchasing and drinking age, and a full ban of alcohol advertisements led to a decrease in road traffic injuries over time [34].

	Facilitate access to screening, brief interventions, and treatment	Evidence indicates that imparting brief advice within primary care settings is a successful intervention to reduce alcohol use and prevent or mitigate progression to alcohol use disorder and addiction. Behavioural and pharmacological therapies have shown to be effective treatments for alcohol use disorder.	A randomized control trial conducted in South Africa showed a positive effect of alcohol screening and intervention using health education leaflets at the beginning of anti-tuberculosis treatment in primary care setting [35]. In the Russian Federation, the Federal Law on Healthcare includes the provision of primary specialised care to people at risk of alcohol abuse, as well as those who indulge in harmful use of alcohol. The law was amended to include a focus on both prevention and treatment. With the recent revision of the law in 2019, the country offers ‘narcology’ specialists in primary healthcare facilities and strict anonymity to patient [36].

	Enforce bans or comprehensive restrictions on alcohol advertising, sponsorship, and promotion	Restricting alcohol advertisement is important to decrease the incidence of alcohol use, considering the impact of these advertisement on adolescents and young people. Advertising bans will prevent adolescents and young adults from being exposed to alcohol and will prevent the alcohol industry from influencing social norms through wrongful depictions in their advertisements.	France’s Loi Evin (Evin’s Law) is a partial ban and includes comprehensive regulation of alcohol advertising, promotion, and sponsorship [37]. Finland is one of the first countries to ban alcohol advertisements on social media [33].

	Raise prices on alcohol through excise taxes and other fiscal policies	Increasing taxes on harmful substances increases their selling cost and subsequently decreases the affordability of such substances. Increasing taxes has been shown to be strongly associated with decrease in alcohol consumption and alcohol-related harms.	In South Africa, the taxation structure has changed from unitary taxes (based on the volume of the alcoholic beverage) to specific volumetric taxes (based on the ethanol content of the alcoholic beverage). As a result, there has been a shift in advertising to low-alcohol beers as they become more profitable to produce [38].

**Other Good Practices**	Establish and enforce a uniform minimum legal drinking age	Increase in legal age to drink alcohol has been found to lead to less drinking in adolescence and subsequently moderate drinking patterns and less frequent harmful drinking patterns as adults.	The increase in the minimum legal drinking age in the USA has been associated with reduced suicide mortality and reduced night-time road traffic fatalities among 18–20 year olds by 17% [39].

	Mandate prominent health warnings on alcohol products	Studies have shown that putting health warnings on alcohol products was associated with an increase in perceptions of health risks of consuming alcohol, as well as greater intentions to reduce and quit alcohol consumption.	The Eurasian Economic Union’s technical regulation mandates provision of an ingredients list, health information, and an additional message of ‘recommendatory nature’ to be put on all types of alcoholic beverages intended for human use [40].

